# Analog-to-Information Conversion with Random Interval Integration

**DOI:** 10.3390/s21103543

**Published:** 2021-05-19

**Authors:** Ján Šaliga, Ondrej Kováč, Imrich Andráš

**Affiliations:** 1 Department of Electronics and Multimedia Communications, Faculty of Electrical Engineering and Informatics, Technical University of Kosice, 040 01 Kosice, Slovakia; imrich.andras@tuke.sk; 2 Department of Technologies in Electronics, Faculty of Electrical Engineering and Informatics, Technical University of Kosice, 040 01 Kosice, Slovakia; ondrej.kovac@tuke.sk

**Keywords:** random interval integration, compressed sensing, analog-to-information conversion, sub-Nyquist sampling

## Abstract

A novel method of analog-to-information conversion—the random interval integration—is proposed and studied in this paper. This method is intended primarily for compressed sensing of aperiodic or quasiperiodic signals acquired by commonly used sensors such as ECG, environmental, and other sensors, the output of which can be modeled by multi-harmonic signals. The main idea of the method is based on input signal integration by a randomly resettable integrator before the AD conversion. The integrator’s reset is controlled by a random sequence generator. The signal reconstruction employs a commonly used algorithm based on the minimalization of a distance norm between the original measurement vector and vector calculated from the reconstructed signal. The signal reconstruction is performed by solving an overdetermined problem, which is considered a state-of-the-art approach. The notable advantage of random interval integration is simple hardware implementation with commonly used components. The performance of the proposed method was evaluated using ECG signals from the MIT-BIH database, multi-sine, and own database of environmental test signals. The proposed method performance is compared to commonly used analog-to-information conversion methods: random sampling, random demodulation, and random modulation pre-integration. A comparison of the mentioned methods is performed by simulation in LabVIEW software. The achieved results suggest that the random interval integration outperforms other single-channel architectures. In certain situations, it can reach the performance of a much-more complex, but commonly used random modulation pre-integrator.

## 1. Introduction

Since its introduction in [1], compressed sensing (CS) has become a recognized method of sub-Nyquist sampling. Rather than acquiring signals at a Nyquist rate, CS relies on a process called analog-to-information conversion (AIC) to capture just enough information for successful signal recovery. Acquiring signals with a lower sampling rate is beneficial in applications where current ADCs are not fast enough [2] or where the sampling itself is energy-demanding [3]. 

CS is often regarded purely as a data processing method applied in discrete spaces. In such cases, the AIC is performed by mathematical matrix multiplication, resulting in a lower number of samples. Options in such cases are virtually limitless, as witnessed by a large number of publications [4,5,6,7,8,9,10,11,12] concerning various applications. On the other hand, when the acquisition of an analog signal has to be performed by physical circuitry, the conditions are more challenging [13]. The AIC circuitry should be as simple as possible, and it has to be describable with sufficient precision by a known matrix. A relatively small number of analog AIC architecture have been proposed. The non-uniform sampler (NUS) [14,15] is among the easiest and cheapest to implement, but has limited versatility. A random demodulator (RD) [16,17,18] is also one of the simpler architecture, but in contrast to NUS, it requires specialized circuitry. The random modulation pre-integrator (RMPI) [19,20,21,22,23] is a multichannel version of RD. It is a widely used versatile wide-bandwidth AIC. Its drawback is high complexity and cost due to its parallelized structure. There have been attempts at simplification of the RMPI circuitry; for example, by compressive circulant matrix AIC [24], Nyquist folding receiver [25], or modulated wideband converter [26,27,28,29]. These still use high-speed analog multipliers, which have been shown to introduce considerable model errors [30,31,32]. The quadrature AIC [33,34,35] relaxes the high-speed front-end requirement, but at the cost of added complexity elsewhere. One of the newest AIC architecture, the non-uniform wavelet sampling [36] is promising in that it is not inherently parallelized. Another non-uniform wavelet bandpass sampling approach uses analog integration of the measured signal, which is multiplied with wavelets functions [37,38]. It can be seen as NUS, which is susceptible only to jitter error [39], with added filter in front. It is again a specialized circuitry that complicates implementation and can introduce model errors.

Most of the listed AIC architecture cannot be built using off-the-shelf components. Custom modules, FPGAs, or even ASICs are required. This hinders the wide adoption of CS and complicates early phases of even experimental CS system development. In this paper, the authors propose and study a new AIC method, the random interval integration (RII). It is aimed at low cost and simplicity, while providing competitive performance. Besides logic circuitry required by any AIC method, the RII requires only a fast integrator and a switch. This simple structure not only eases implementation, but also minimizes room for modeling errors.

The paper is organized as follows. Section 2 provides an overview of CS. Section 3 describes the proposed RII. Performance is evaluated in Section 4 by means of simulations, where the RII is compared to previously proposed AIC methods. In Section 5, an RII prototype is presented and tested. The paper is concluded in Section 6.

## 2. Compressed Sensing Overview

CS can be applied if the input signal is expressible as a vector $f\in R^{N\times 1}$, which is compressible and can be classified as sparse. Let there exist a set $\Psi \in R^{N\times L}$ of basis functions $\psi_{l}\in R^{N\times 1},1\leq l\leq L$, such that any possible input signal can be described as:

(1) f=Ψx.

Vector $x\in R^{L\times 1}$ performs a linear combination of the basic functions. If x has only *s* entries of appreciable value and s<<L, the signal is denoted as *s*-sparse. This signal can be sampled by correlating it with M measurement signals, M<N, defined by the measurement matrix $\Phi \in R^{M\times N}$. Resulting is the signal:

(2) $$y=\Phi f\in R^{M\times 1},$$

where y has fewer samples (lower sampling frequency) than f but contains all the information needed for reconstruction. y is hence referred to as the information signal. The measurement matrix usually has random attributes, so aliasing is avoided. If the matrix Φ is subject to implementation on a physical circuitry, entry values of 0 and ±1 associated with switches are preferred.

In the reconstruction phase, only bases Ψ, Φ and the information signal y are known. Let

(3) $$A=\Phi \Psi \in R^{M\times L},$$

be denoted as the reconstruction matrix for convenience. Estimate of the original signal

(4) $$\hat{f}=\Psi \hat{x},$$

can be obtained after finding $\hat{x}$ by solving

(5) $$\min∥\hat{x}{∥}_{p}\mathrm{subject}\;\mathrm{to}\;A\hat{x}=y,\;p=0,\;1\;\mathrm{or}\;2.$$

It is the task of the analog-domain AIC to go from an analog input signal $f\left(t\right)$ straight to y without ever sampling into f. The behavior of AIC circuitry during this process is modeled by Φ, which must be done with sufficient precision. For example, an idealized NUS can be described by Φ with entries

(6) $$\phi_{mn}=\left\{\begin{array}{c} 1\\ 0 \end{array}\right.\begin{array}{c} \mathrm{if}\;n=\xi_{m}\\ \;\mathrm{otherwise} \end{array},$$

where $\xi_{m}\in 1;N,\;m=1,2,\ldots ,M$ is the random sampling sequence. With a real NUS, modeling error would be caused by clock jitter and nonideal ADC. Additional modeling errors are present with more complex architecture. These are undesirable because of their impact on the reconstruction error.

## 3. Random Interval Integration

**Figure 1:**
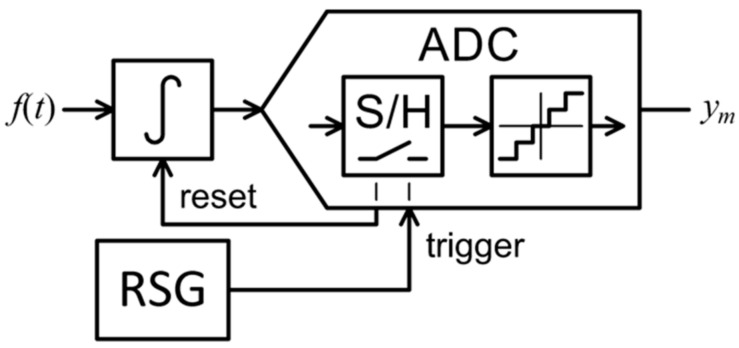
Random interval integration analog-to-information converter.

The authors propose the random interval integration (RII) AIC architecture shown in Figure 1 with practical implementation presented in Section 5. A continuous input signal is integrated for a random period by a resettable integrator. After this period has passed, a conventional ADC samples the integrator output, obtaining a sample of the information signal. The integrator is then immediately reset and continues to integrate the input signal. Random integration times are defined by a random sequence generator (RSG). Using the random sampling sequence from (6), idealized RII measurement matrix can be described as:

(7) $$\phi_{mn}=\left\{\begin{array}{c} 1\\ 0 \end{array}\right.\begin{array}{c} \mathrm{if}\;\xi_{m-1}<n\leq \xi_{m}\\ \;\mathrm{otherwise} \end{array}.$$

Equation (7) assumes that the time for which the integrator is being reset—and the input is not being integrated—is negligible. If this time is equivalent to k samples of ϕ, (7) can be modified as follows:

(8) $$\phi_{mn}=\left\{\begin{array}{c} 1\\ s\left(n,m\right) \end{array}\right.\begin{array}{c} \mathrm{if}\;(\xi_{m-1}+k)<n\leq \xi_{m}\\ \;\mathrm{otherwise} \end{array}.$$

$s\left(n,m\right)=0$ for simplification or it can be a suitable function that describes the switching transient.

The proposed RII architecture is intended mainly for acquisition of aperiodic or quasiperiodic signals. The information is collected from the input signal during the entire integration period. This increases the chance of capture and reconstruction of aperiodic signal features, provided that these are included in the dictionary Ψ. Signals where such artifacts are common are, for example, ECG [11] or water parameter signals (WPS) [3].

**Figure 2:**
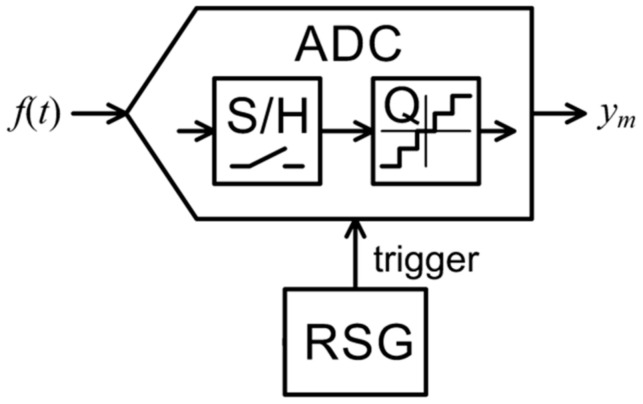
Nonuniform sampling analog-to-information converter.

There is a high risk of missing aperiodic signal features with NUS [14], shown in Figure 2. There are periods when no sample is taken and thus critical information can be left out of y. NUS is commonly tried in all sorts of applications because of its simplicity, but it is best suited for periodic signals with no non-repeating artifacts that can be missed.

The drawback of integration is that the integrated value can be small or 0 if input signal is periodic. This is a form of aliasing, which can occur if the integration period is close to the integer multiple of input signal frequency. If the integrated value is 0, information is missed; if it is a small value, then quantization may introduce significant errors. The RII therefore introduces randomization of the integration period, which is intended to lower the risk of such occurrences for periodic signal components.

**Figure 3:**
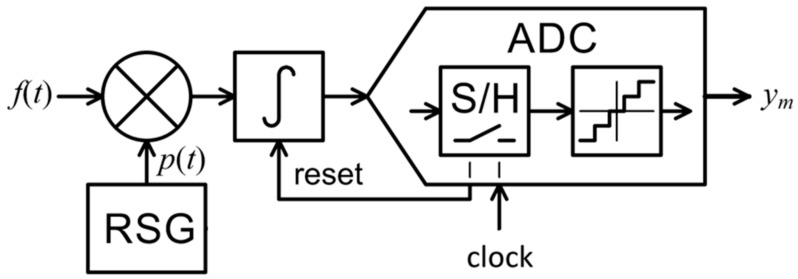
Random demodulation analog-to-information converter.

The RD’s (shown in Figure 3) multichannel version RMPI and other AIC architecture also employ randomization, though it is performed right at the front-end at Nyquist rate. Samples are then taken at a constant low rate. The specialized front-end multiplier requires fast switches or a linear mixer circuit for its implementation [32]. The proposed RII in contrast does not have a multiplier at the front-end, introducing randomization solely via trigger control. The resettable integrator allows to use a relatively slow switch, since it is situated towards the output where signal is already low bandwidth. The only component that has to handle the fast input signal in RII is the integrator. This is a technologically mature and commonly available part so the amount of specialized circuitry in RII AIC is minimized.

## 4. Experimental Results

The performance of the proposed RII method was evaluated using simulations as well as measurements on a physical RII sample circuit. With simulations, three classes of test signals were used: ECG, multi-sine, and water parameter signals (WPS) [3]. The selected types of test signals were sampled by simulated converters. The simulated acquisition used AIC with NUS, RD, RMPI, and the proposed RII. Idealized AIC models were used, such as (6) and (7). Performance with a varied compression ratio

(9) $$C=\frac{N}{M},$$

noise added to samples and sample quantization were tested. Under each set of test conditions, 100 runs of sampling and reconstruction were performed. A new measurement matrix was generated for every run and the results were averaged. The AIC performance was evaluated by reconstruction quality, enumerated as a signal-to-deviation ratio (SDR) [3]:

(10) $$SDR=10log\left(\frac{\sum_{n=1}^{N}{\hat{f}}_{n}^{2}}{\sum_{n=1}^{N}{\left(f_{n}\;-\;{\hat{f}}_{n}\right)}^{2}}\right)\left[\mathrm{dB}\right].$$

The ECG signals used for testing were generated using the LabVIEW biomedical toolkit. Signals representing 9 heart conditions with 500 random parameter variations for each diagnosis were used, for a total of 4500 test signals. Each ECG test signal contained one heart cycle with n=360. The dictionary Ψ was extracted using principal component analysis [40]. The signals were reconstructed using orthogonal matching pursuit [41].

Multi-sine test signals contained three sine components with random amplitudes, frequencies, and phases. Amplitudes ranged within ±1, phases within ±π/2 and frequencies between 0.1$f_{s}$, and 0.2$f_{s}$, where $f_{s}$ is the reconstruction sampling frequency. A sine-cosine dictionary [42] with 0.075$f_{s}$–0.225$f_{s}$ range and 2.5$\times 10^{-3}f_{s}$ resolution was used for reconstruction. A total of 120 multi-sine test signals with n=120 were used.

The WPS are real-world signals representing the chemical properties of a freshwater stream [3]. 120 WPS with n=120 were used for testing with reconstruction based on principal component analysis.

### 4.1. Compression Ratio

**Figure 4:**
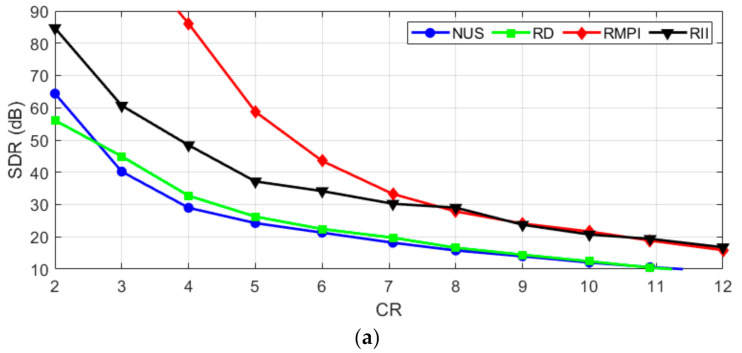

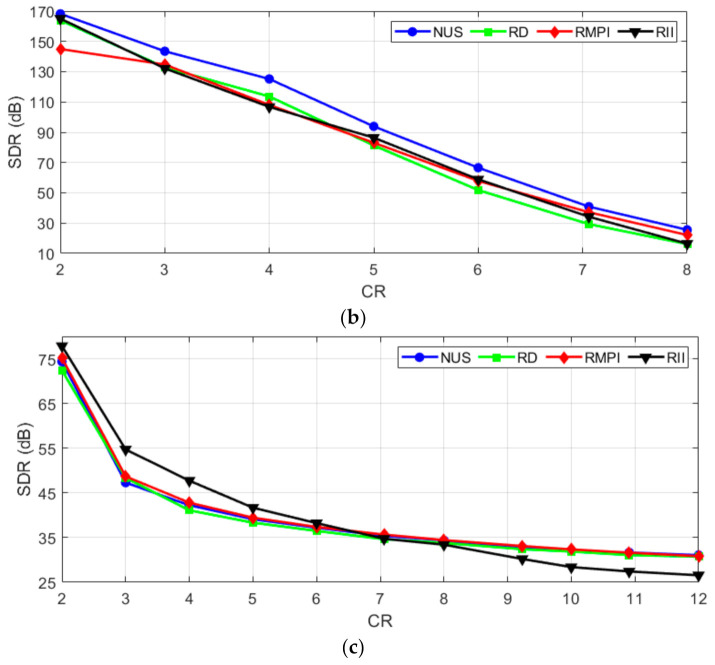
Reconstruction with varying CR for (**a**) ECG; (**b**) Multi-sine; (**c**) WPS signal.

CR is one of the key parameters that have to be chosen during the design of a CS system. The following figures show how the AIC methods influence reconstruction when sampling a certain type of signal with given CR. The values of measured SDR achieved by the analyzed method applied to the test signals are shown in Figure 4.

Figure 4a shows that NUS and RD are not very suitable for acquiring ECG. The expensive RMPI performs best, as was expected. The proposed RII is in the middle, which is a positive result, considering its simplicity. Furthermore, the RII is even comparable with RMPI at higher compression ratios. Based on Figure 4b, it can be concluded that NUS seems to be a method of choice for acquiring multi-sine signals. It is the simplest and at the same time, the best performing AIC method. RII performs approximately the same as RD and RMPI. Figure 4c shows that on acquiring of WPS, the three reference methods perform almost the same across the board. The proposed RII performs slightly better with low CR < 7 and slightly worse with higher CR. From the charts, it can be concluded that the best SDR, in general, is achieved on multi-sine signals. This comes from the periodical character and high sparsity level of these signals.

### 4.2. Additive Noise

**Figure 5:**
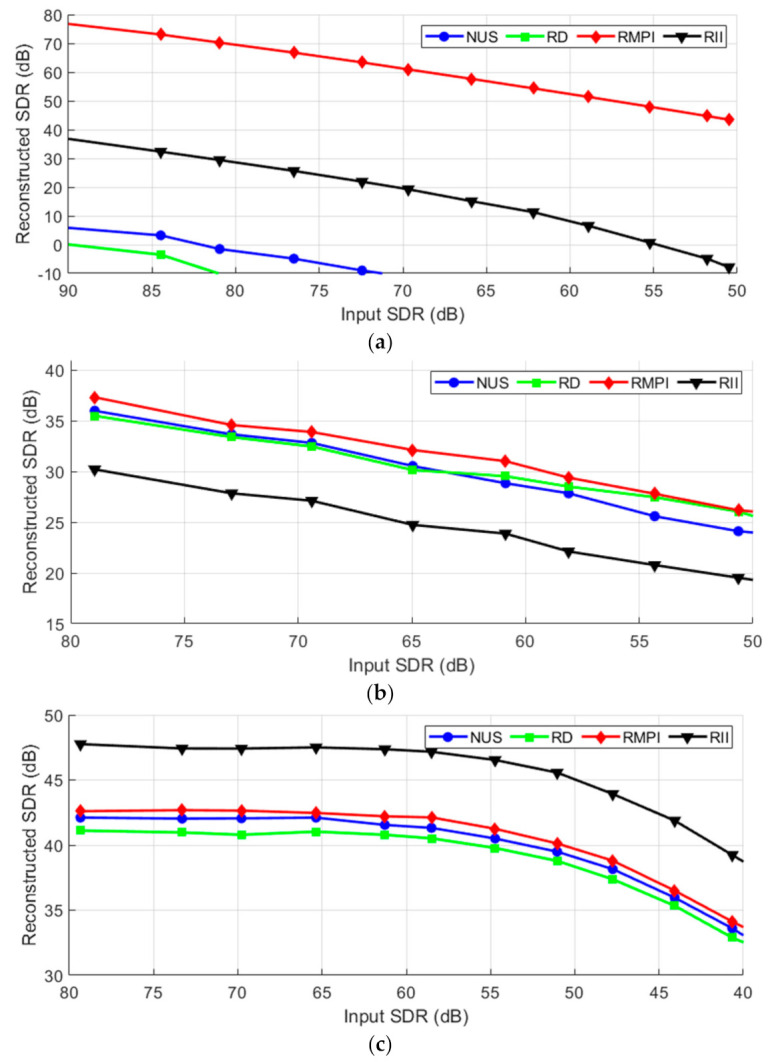
Reconstruction for noised (**a**) ECG; (**b**) Multi-sine; (**c**) WPS signal.

Figure 5a shows noisy ECG reconstruction with RMPI and RII, which is consistent with Figure 4a. The descent of the SDR of the reconstructed signal for RMPI and RII is almost linear with the increase of the input SDR. Despite being a single channel architecture, RII resistance to noise is similar to that of RMPI. The other single-channel architecture—NUS and RD—seem to amplify the effects of noise; adding even a small amount causes a significant SDR decrease. Figure 5b shows that the proposed RII is not particularly suitable for acquiring frequency sparse signals. When adding noise to multi-sine signals, the results differ from those under ideal conditions (Figure 4b). The reference methods perform approximately the same, but RII performance suffered with the introduction of noise. On the other hand, the proposed RII performs the best when acquiring noisy WPS (Figure 5c), which is consistent with results under ideal conditions (Figure 4c). It can be concluded that acquiring WPS signals is the most resilient to additive noise, it has no significant influence on reconstruction quality until approx. 55dB, when reconstructed SDR starts dropping significantly.

### 4.3. Sample Quantization

**Figure 6:**
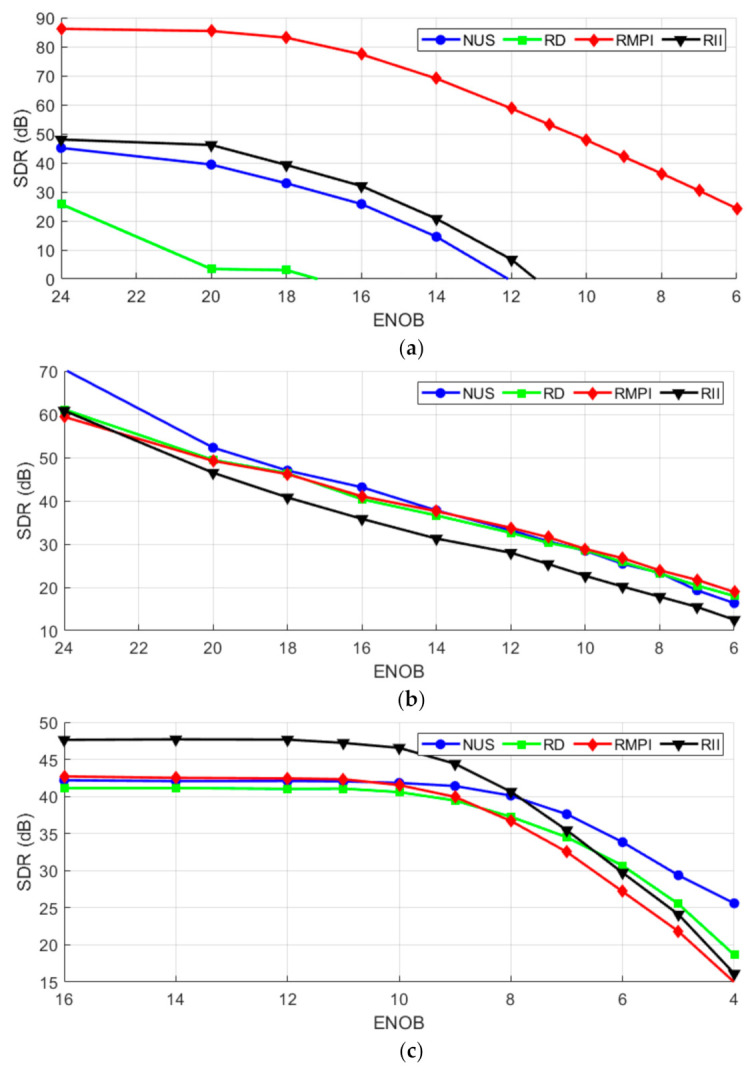
Reconstruction with varying ENOB for (**a**) ECG; (**b**) Multi-sine; (**c**) WPS signal.

ADC resolution is another of the key parameters that must be taken into consideration during the CS system design. Figure 6 shows how quantization noise influences reconstruction with CR = 4. ADC resolution is denoted by the effective number of bits (ENOB). The results shown in Figure 6 are very similar to the results from the previous experiment. Though the quantization noise has a different nature than additive noise, it is still a noise considering dictionary Ψ. The results of RII are consistent with those from the previous subchapter. RII seems to be sensitive to noise when sampling multi-sine, though not as much with quantization as it is with random noise.

### 4.4. Demonstration and Discussion

**Figure 7:**
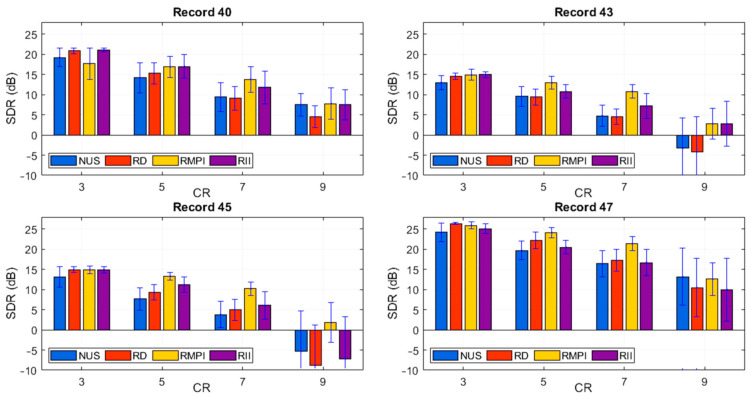
The dependence of SDR on the CR for ECG records #40, #43, #45, and #47.

The next experiment is focused on the ECG signal reconstruction. It was performed on one period of four ECG records (#40, #43, #45, and #47). The signals were obtained from the MIT-BIH database and the dictionary was created on the basis of 40 different records from the database. In Figure 7, SDR dependence on the CR for all analyzed methods is shown. The results indicate that the proposed method is comparable with other analyzed methods.

**Figure 8:**
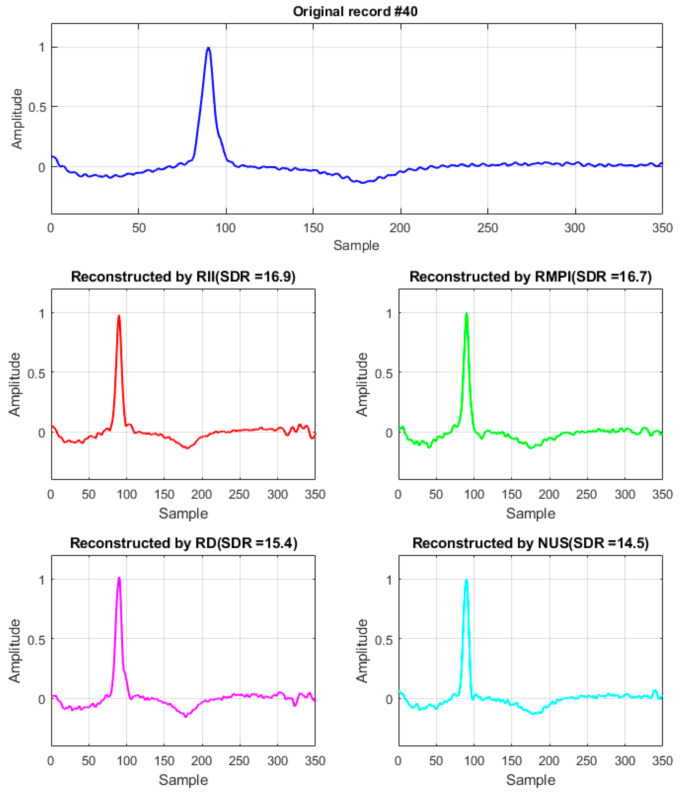
The ECG record #40 of the MIT-BIH database and its reconstruction for CR = 5.

For better illustration of the reconstruction quality achieved by all mentioned methods, in Figure 8, the record #40 for CR = 5 is shown.

**Figure 9:**
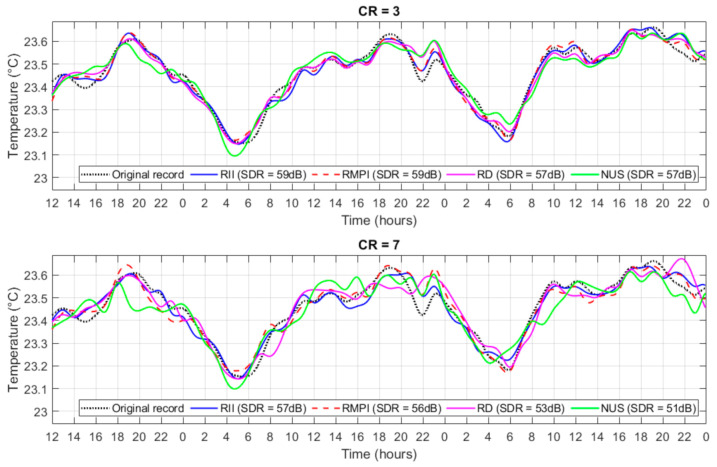
The example of the WPS signal (water temperature) and its reconstruction for CR = 3 and 7.

The signals contained in WPS were of various origins. These parameters are commonly measured in water: temperature, conductivity, salinity, PH, oxygenation, and redox. In Figure 9, the temperature record from the probe placed in a river for a period of three days is shown. The simulation was performed for all analyzed methods and for two values of CR (3 and 7). From the charts, in Figure 9 it can be concluded that for CR = 3, the reconstruction for all analyzed methods is highly correlated with original measures. However, the NUS had worse reconstruction than the other methods. The proposed RII is comparable or slightly better than RMPI. If CR = 7, the result of reconstruction is significantly worse. The RD and NUS are significantly deviant from the original data. The inaccuracy of the reconstruction achieved by RII and RMPI appears mainly in parts where the change of the measured value is rapid. These results are comparable with the results shown in Figure 4. The proposed RII is highly suitable for CS of WPS signals and mainly for lower CR.

**Figure 10:**
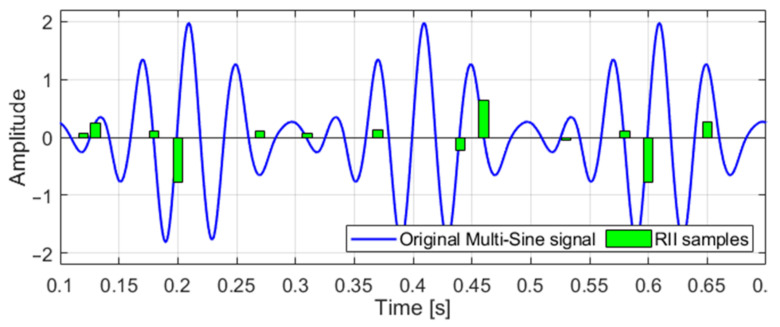
The example of multi-sine signal and its RII compressed version.

An example of the multi-sine signal is shown in Figure 10, where the original analog signal and its compressed version are shown. The green bars represent an integrated value of the compressed version of the signal. In this case, the multi-sine signal consists of frequencies 0.2, 0.25, and 0.3 Hz. The CR is equal to 5. It is possible to show that the reconstructed signal because of very high SDR for low CR is almost identical to the input signal. Thus, the reconstruction is not present in the charts.

## 5. Hardware Implementation

A multi-sine signal analyzed in previously mentioned simulations was chosen for hardware implementation. For this purpose, amplitude modulation (AM) that can be easily generated was chosen. The AM signal consists of a carrier frequency and two sidebands. The AM test signals are known for their frequency-sparse structure.

**Figure 11:**
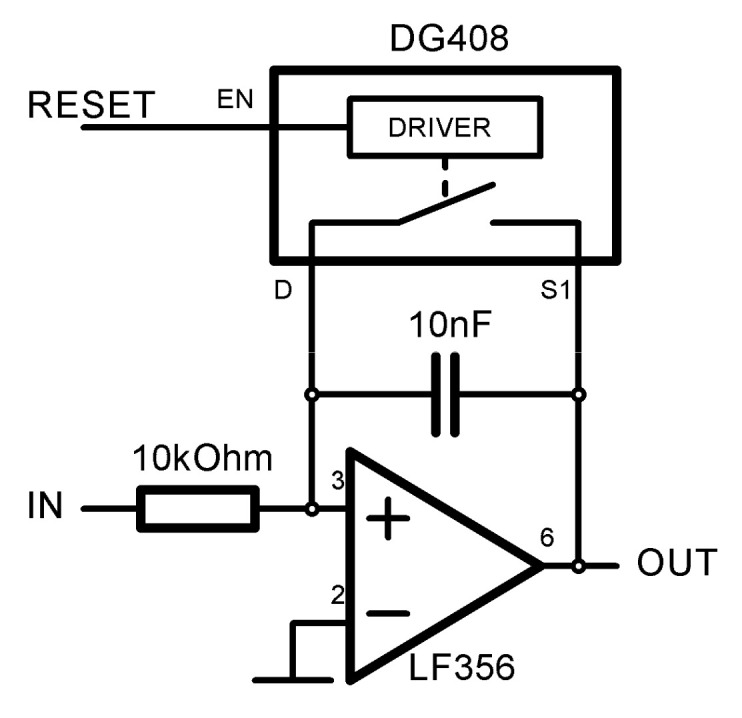
Analog part of the RII test prototype.

The feasibility of the proposed RII architecture was demonstrated by a prototype. A schematic of its analog part is shown in Figure 11. The circuit is consistent with the previously mentioned block diagram in Figure 1. The integrated circuit LF356 with a capacitor of 10 nF and 10 kΩ resistor is used as a hardware integration circuit. The integration reset is performed by shorting the capacitance using the switch in analog multiplexer DG408. Any sufficiently fast switch with low parasitic capacitance can be used for this purpose. The reset is triggered externally by myRIO-1900 FPGA. In general, the reset can be triggered by an arbitrary circuit or microprocessor, but the myRIO offers its programming with LabVIEW and provides the FPGA high precision random timing solution. The myRIO is used as an ADC as well as an interfacing device with PC and LabVIEW software.

**Figure 12:**
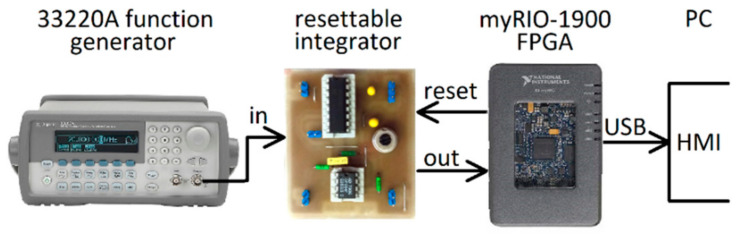
Measurement setup with the RII prototype.

The RII prototype comprised of the circuit in Figure 11 and an FPGA with RSG and ADC onboard. The final prototype is shown in Figure 12.

**Figure 13:**
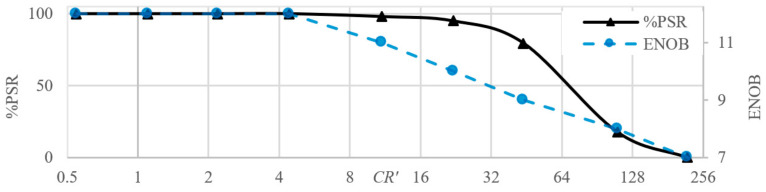
The results achieved for constant AIC setup and varying input signal frequency.

The original signal was not sampled. Therefore, the probability of successful reconstruction (PSR) was used as a measure of success. The signal was considered successfully reconstructed if its frequency components matched those of the input signal with 1% tolerance for frequency and 10% tolerance for magnitude. False frequency components were allowed if their magnitude was less than 2% of the carrier. Figure 13 shows the results for constant AIC setup and varying input signal frequency.

The following definition of compression ratio is adapted:

(11) $$CR^{\prime }=f_{Im}/f_{Sa},$$

where $f_{Im}$ is the carer frequency within input signal bandwidth and $f_{Sa}$ is the average sampling frequency after RII. The carrier frequency was from interval 500 Hz to 200 kHz and the modulation frequency was 10% of carrier frequency. During the tests, $f_{Sa}$ was 1 kHz with reliable reconstruction up to $f_{Im}=50\;\mathrm{kHz}$. (CR = 50, PSR = 80% in Figure 13). The modulation depth was always set to 50%. This range could be shifted lower or higher by changing the RC constant and widened by using a higher resolution ADC. Commercially available fast amplifiers and switches could bring RII into the GHz range.

The integrator acts as a low-pass filter; therefore, the integrated values decrease with increasing frequency. This decrease of amplitude effectively lowers the ENOB, which is also shown in Figure 13. The practical bandwidth of RII is thus determined by the RC constant but also ADC resolution. Assuming a constant $f_{Sa}$, a lower RC constant allows a higher bandwidth but raises the risk of saturating the integrator if low frequency components are present in the input signal. Higher RC constant allows to measure low frequency signals, but high enough ENOB is required to also acquire high frequency components, which will become low amplitude.

## 6. Conclusions

The random interval integration, a novel method of analog-to-information conversion, is presented in this paper. Simulations show that this method, in many cases, outperforms previous single-channel architecture, while being similar or even less complex. It was demonstrated that in certain situations, the proposed RII can reach and even outperform the performance of RMPI, considered as state-of-the-art, but a much more complex architecture. The best results were achieved on WPS signals; however, on the other hand, the proposed RII is not very suitable for multi-sine signals, where the simpler NUS method performed better. The results achieved by RII applied on ECG signals are promising because the RII is much simpler than RMPI and the performance is comparable when a higher compression ratio is used.

RII is a low-cost AIC method worth considering in general, and particularly suitable for acquiring aperiodic signals such as ECG and WPS. The proposed method provides competitive performance, while preserving low cost and circuit simplicity.

## Data Availability

Not applicable.
